# Cerebrospinal fluid biomarkers of axonal and synaptic degeneration in a population-based sample

**DOI:** 10.1186/s13195-023-01193-x

**Published:** 2023-03-03

**Authors:** Maya Arvidsson Rådestig, Ingmar Skoog, Tobias Skillbäck, Henrik Zetterberg, Jürgen Kern, Anna Zettergren, Ulf Andreasson, Hanna Wetterberg, Silke Kern, Kaj Blennow

**Affiliations:** 1 Department of Neuropsychiatric Epidemiology Unit, Institute of Neuroscience and Physiology, Sahlgrenska Academy at the University of Gothenburg, Gothenburg, Sweden; 2 Department of Psychiatry and Neurochemistry, Institute of Neuroscience and Physiology, Sahlgrenska Academy at the University of Gothenburg, Gothenburg, Sweden; 3 Present Address: Department of Psychiatry, Cognition and Old Age Psychiatry, Sahlgrenska University Hospital, Gothenburg, Sweden; 4 Clinical Neurochemistry Laboratory, Institute of Neuroscience and Physiology, Sahlgrenska Academy at the University of Gothenburg, Mölndal, Sweden; 5 Department of Neurodegenerative Disease, UCL Institute of Neurology, Queen Square, London, WC1N 3BG UK; 6 UK Dementia Research Institute at UCL, London, WC1N 3BG UK; 7 Hong Kong Center for Neurodegenerative Diseases, Hong Kong, China

**Keywords:** Alzheimer’s disease, Cerebrospinal fluid, CSF, biomarkers, Neurolfilament light, NfL, Neurogranin

## Abstract

**Background:**

Neurofilament light (NfL) and neurogranin (Ng) are promising candidate AD biomarkers, reflecting axonal and synaptic damage, respectively. Since there is a need to understand the synaptic and axonal damage in preclinical Alzheimer’s disease (AD), we aimed to determine the cerebrospinal fluid (CSF) levels of NfL and Ng in cognitively unimpaired elderly from the Gothenburg H70 Birth Cohort Studies classified according to the amyloid/tau/neurodegeneration (A/T/N) system.

**Methods:**

The sample consisted of 258 cognitively unimpaired older adults (age 70, 129 women and 129 men) from the Gothenburg Birth Cohort Studies. We compared CSF NfL and Ng concentrations in A/T/N groups using Student’s *T*-test and ANCOVA.

**Results:**

CSF NfL concentration was higher in the A−T−N+ group (*p*=0.001) and the A−T+N+ group (*p*=0.006) compared with A−T−N−. CSF Ng concentration was higher in the A−T−N+, A−T+N+, A+T−N+, and A+T+N+ groups (*p*<0.0001) compared with A−T−N−. We found no difference in NfL or Ng concentration in A+ compared with A− (disregarding T− and N− status), whereas those with N+ had higher concentrations of NfL and Ng compared with N− (*p*<0.0001) (disregarding A− and T− status).

**Conclusions:**

CSF NfL and Ng concentrations are increased in cognitively normal older adults with biomarker evidence of tau pathology and neurodegeneration.

## Introduction

Alzheimer’s disease (AD) is a progressive neurodegenerative disease characterized by intracerebral accumulation of amyloid (Aβ) and abnormally phosphorylated tau, followed by neurodegeneration leading to progressive cognitive decline. Cerebrospinal fluid (CSF) amyloid β42 (Aβ42), total tau (T-tau), and phosphorylated tau (P-tau) concentrations are established diagnostic and/or prognostic biomarkers for AD, reflecting the core pathological hallmarks of AD, amyloid plaques, neurodegeneration and the hyperphosphorylation of tau, respectively [1]. In recent years, two new promising biomarkers for axonal and synaptic degeneration in AD, neurofilament light protein (NfL) and neurogranin (Ng) have emerged. Ng is a post-synaptic protein that is enriched in dendritic spines expressed primarily in the cortex and hippocampus [2]. CSF Ng concentrations have been shown to be increased in the pre-dementia or mild cognitive impairment (MCI) stages of AD [3–5] and seem to be specifically increased in AD [6]. Loss of synapses has been shown to be an early event in the development of AD [5, 7–9], and dysfunction and loss of synapses are thought to precede neurodegeneration [4, 6]. NfL is an axonal protein that is released to the CSF following damage to particularly large caliber myelinated axons [10, 11]. CSF NfL is a biomarker that reflects neuronal damage irrespective of cause, while CSF Ng reflects synaptic damage in AD [12], but data from population-based studies in the cognitively unimpaired are rare [13, 14]. There is a need to understand the very early preclinical stages of AD better in terms of axonal and synaptic degeneration. We therefore aimed to assess CSF NfL and CSF Ng levels in cognitively unimpaired elderly from the Gothenburg H70 Birth Cohort Studies classified by CSF biomarker concentrations according to the A/T/N system.

## Method

The sample was derived from the 2014–2016 examinations of the population-based H70 Gothenburg Birth Cohort Studies in Gothenburg, Sweden. Residential addresses were obtained from the Swedish Population Registry. The sample was obtained using systematic selection, where every 70-year-old living in Gothenburg (households and residential care), born on specific dates in 1944 was invited to partake in examination in 2014–2016 [15]. 1203 subjects opted to participate (response rate 72.2%). Four hundred thirty of these individuals (35.8%) consented to a lumbar puncture (LP). Contraindications to LP (anticoagulant therapy, immune modulated therapy, cancer therapy) were present in 108, and extracted CSF volumes were insufficient for proper analysis in four participant samples, leaving 318 tested subjects in the final sample (26.4%) [15, 16]. We defined participants as cognitively normal if they had a global clinical dementia rating (CDR) score [17] of 0, leaving 258 participants with NfL and Ng data for the current study. Four participants were removed because they had extreme NfL levels (>3.5 SD from the mean). The first excluded participant had a CSF NfL concentration of 4976 and a CSF Ng concentration of 184 and was classified as A−T−N−. The other three excluded participants had a history of cancer which may affect NfL levels [18]. Their CSF NfL concentrations were 12312, 6122, and 6056 pg/mL, their CSF Ng concentrations were 352, 132, and 299 pg/mL, and they were classified as A−T+N+, A−T−N−, and A−T−N+. All participants and/or their close relatives gave written informed consent to participate in the study. The study was approved by the Regional Ethical Review Board in Gothenburg (Approval number 869-13).

### Examinations

Participants took part in a full-day examination at the Psychiatry, Cognition, and Old Age outpatient clinic at Sahlgrenska University Hospital in Gothenburg, Sweden, or in their homes as described previously [15].

A comprehensive general examination was performed that included blood sampling and genotyping, use of medications, self-rating questionnaires, social factors, key informant interviews, and neuropsychiatric examination [15, 19]. The neuropsychiatric examination, which was performed by psychiatric research nurses, comprised ratings of psychiatric symptoms and signs, tests of mental functioning, including assessments of episodic memory (short-term, long-term), aphasia, apraxia, agnosia, executive functioning, and personality changes [15, 19, 20].

Examinations included the Mini-Mental State Examination (MMSE) and CDR. The final ratings were assigned by research nurses and a geriatric psychiatrist and neurologist (SK). All examinations were performed by trained and experienced research staff. Dementia was diagnosed according to DSM-III-R [21], keeping with protocol as established in the Gothenburg studies since more than 30 years [19].

Stroke and transient ischemic attack (TIA) information was acquired from self-reports and key informants. Education, defined in years of education, was assessed by self-report or key informant information [15, 16].

### Apolipoprotein E (APOE) genotyping

The single nucleotide polymorphisms (SNPs) rs7412 and rs429358 in *APOE* (gene map locus 19q13.2) were genotyped using KASPar® PCR SNP genotyping system (LGC Genomics, Hoddesdon, Herts, UK). Genotype data for these two SNPs were used to define ε2, ε3, and ε4 alleles [16], and *APOE* genotype data was missing for five individuals.

### Lumbar puncture and biomarker analyses

Lumbar punctures (LP) to collect CSF samples were performed in the L3/L4 or L4/L5 inter-space in the morning. The procedure has been described elsewhere [15, 16].

CSF T-tau, P-tau, and Aβ42 were analyzed as part of clinical routine diagnostics [22]. CSF T-tau and P-tau (phosphorylated at threonine 181) were measured using a sandwich enzyme-linked immunosorbent assay (ELISA) (INNOTEST® htau Ag and PHOSPHOTAU (181P), Fujirebio (formerly Innogenetics) [23, 24]. A sandwich ELISA (INNOTEST® β-amyloid_1–42_), specifically constructed to measure the 1–42 isoform of Aβ [25] was used to determine CSF Aβ42. CSF NFL and Ng were stored at −80°C and measured using in-house-developed ELISAs developed at the Mölndal Clinical Neurochemistry Laboratory as described previously [4, 26].

### A/T/N classification

The A/T/N classification scheme was used to classify participants [27]. Participants were classified using 3 binary categories, into 8 possible biomarker combinations. “A” refers to evidence of Aβ pathology (here defined as CSF Aβ42 levels ≤ 530 pg/mL), “T” to evidence of tau pathology (here defined as CSF P-tau ≥ 80 pg/mL), and “N” to evidence of neurodegeneration (here defined as CSF T-tau ≥ 350 pg/mL) [16, 28, 29]. Data on NfL, Ng, and ATN in cognitively unimpaired participants were available in 258 individuals.

### Statistical analysis

The NfL and Ng variables were both skewed to different degrees, and they were both log-transformed (log-10) prior to statistical analysis. Spearman correlation analysis was used to analyze correlations between biomarkers. We also performed ANOVA (analysis of variance) and ANCOVA (ANOVA with covariates) to analyze differences in means of log(NfL) and log(Ng) in the ATN groups. In ANOVA analysis we used ATN groups as an independent variable and log(NfL) level as a dependent variable, without covariates. The same procedure was used for log(Ng). ANCOVA was performed with log(NfL) and log(Ng) as a dependent variable, ATN groups as an independent variable, and the covariates age, sex, stroke, and *APOE ε4* carriership status, including the Bonferroni adjustment for multiple comparisons. We also performed pairwise comparisons between the ATN groups as a post hoc test, and the same covariates were included.

A two-tailed significance level (*p*<0.05) was selected. Statistical analyses were performed in IBM SPSS Statistics for Windows (v. 25, SPSS, Armonk, NY.)

## Results

The 258 participants of this study had a mean age of 70.6 (SD=0.3) years and 129 (50%) were female. Mean education was 13.1 (SD=3.9) years and 86 (33.3%) were heterozygote or homozygote *APOE ε4* carriers. Eight participants (3.1%) had a history of stroke. The median NfL concentration was 724 pg/mL (IQR=384), and the median Ng concentration was 196 pg/mL (IQR=80) [30].

### Correlation between biomarkers

**Fig. 1:**
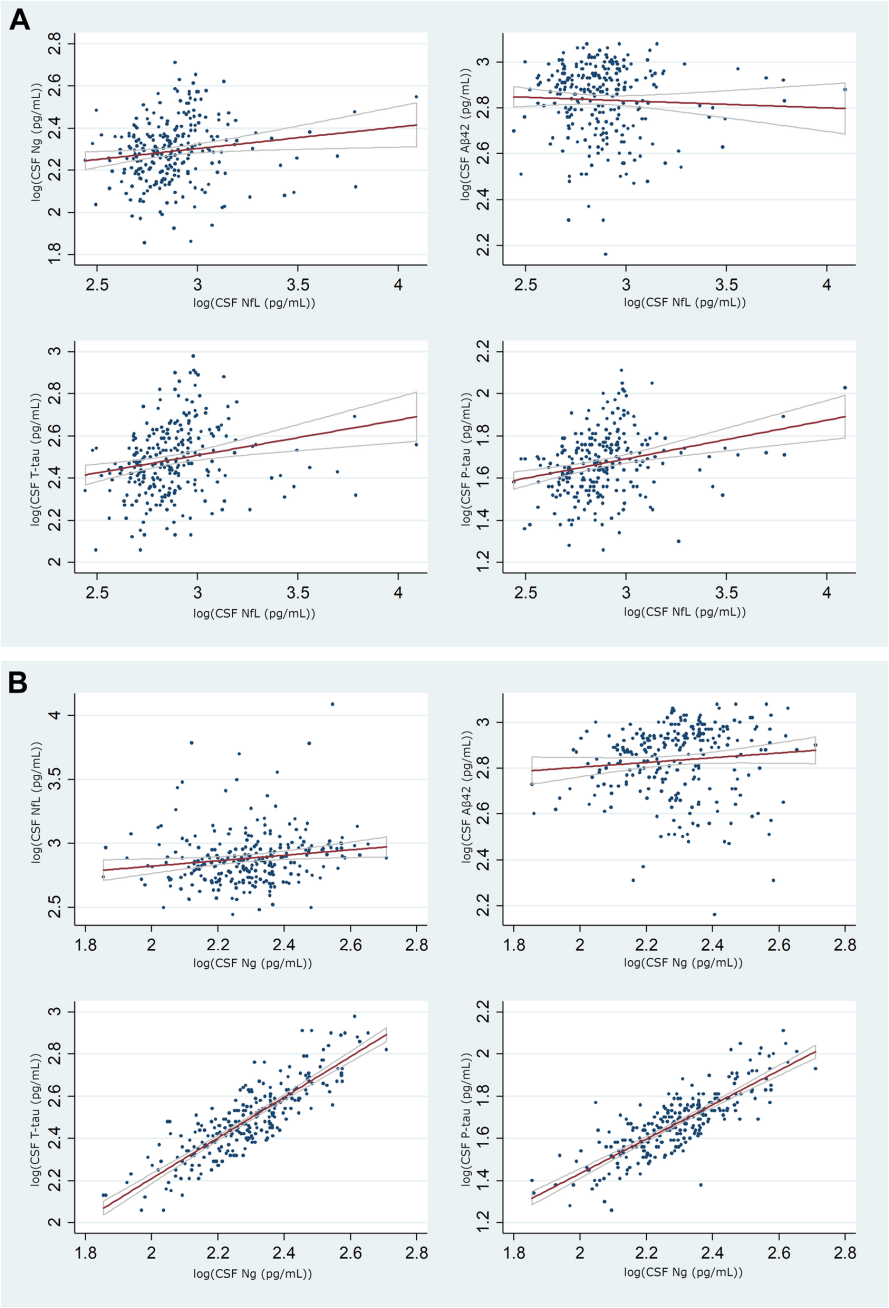
Correlations between biomarkers. **A** NfL correlations. **B** Ng correlations

Correlations between NfL, Ng, T-tau, P-tau, and Aβ42 are displayed in Fig. 1. NfL and Ng correlated weakly with each other (rho=0.21, *p*= 0.001). NfL also correlated with T-tau (rho=0.31, *p*<0.0001) and P-tau (rho=0.31, *p*<0.0001). NfL did not correlate with Aβ_42_ (rho=−0.06 *p*=0.4). Ng correlated strongly with T-tau (rho=0.84, *p*=<0.001) and P-tau (rho=0.81, *p*=<0.001) and weakly with Aβ_42_ (rho=0.19, *p*=0.002). The distribution of the ATN groups among the whole group of cognitively normal participants has been described previously [16, 31].

Our sample with data on CSF NfL and Ng concentrations comprised 258 cognitively unimpaired participants who were distributed among the ATN groups as follows: A−T−N−: 138 (53.5%), A−T−N+: 49 (19.0) A−T+N+: 12 (4.7%) A+T−N−: 33 (12.8 %) A+T−N+: 20 (7.8%) A+T+N+: 6 (2.3%), while there were no participants with A−T+N− and A+T+N− [16, 31]..

Concentrations of CSF-NfL and CSF-Ng in the ATN groups are given in Table 1.

**Table 1:** NfL and Ng concentrations in the H70 study participants stratified by ATN classification

	A−T−N−	A−T−N+	A−T+N+	A+T−N−	A+T−N+	A+T+N+
**Nfl (*N*)**	137	49	12	32	20	6
**NfL (median) pg/ml** **(min/max)**	622.0 (313/6122)	849.0 (481/6056)	899.0 (536/12312)	738.0 (276/3022)	802.5 (483/1886)	959.5 (771/1027)
**NfL-Log (mean) pg/ml**	2.8	3.0	3.0	2.9	2.9	3.0
**Ng (N)**	138	49	12	33	20	6
**Ng (median) pg/ml** **(min/max)**	170.6 (71.7/303.9)	244.7 (179.3/376.4)	350.2 (297.5/512.5)	160.9 (72.9/254.5)	245.9 (185.8/377.5)	338.9 (285.4/410.3)
**Ng-Log (mean) pg/ml**	2.2	2.4	2.6	2.2	2.4	2.5

There were no participants in the A−T+N− and the A+T+N− group [16]

There were no other statistically significant differences.

### Group comparisons

**Fig 2:**
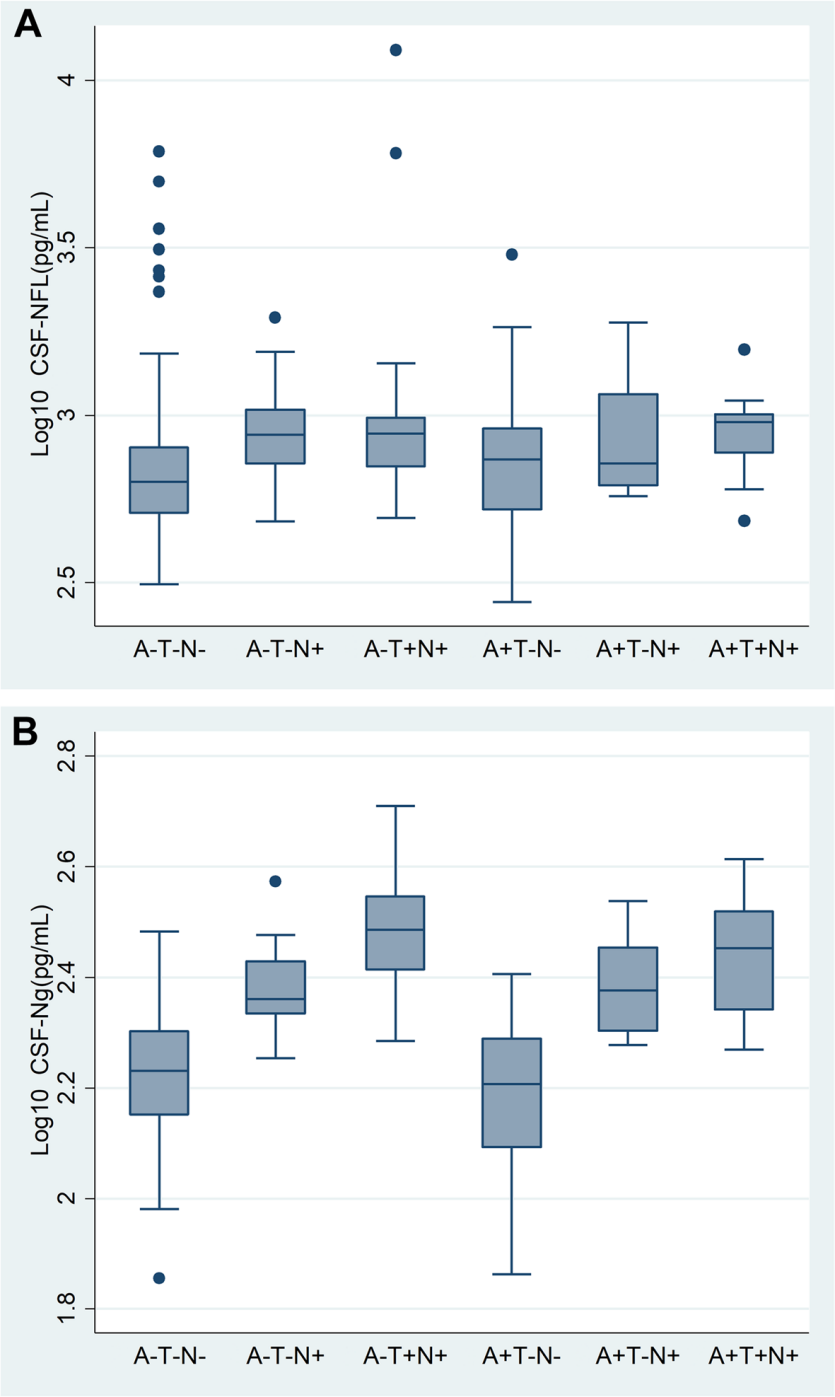
Concentration of NfL and Ng in ATN groups.** A** NfL concentration boxplot. **B** Ng concentration boxplot

When comparing the CSF log(NfL) concentrations in the different ATN groups using t-tests, we found that the groups A-T-N+ (*p*=0.001) and A-T+N+ (*p*=0.006) had higher concentrations than the A-T-N- group (Fig. 2A, Table 2). There were no statistically significant differences among the other groups.

**Table 2:** Group comparisons using log-transformed CSF-NfL and CSF-Ng concentrations in ATN groups in participants with global CDR0 (*n*=258)

Neurofilament light (NfL)					Neurogranin (Ng)			
ATN class	***N***	Mean (SD)	Cohen’s D	***p***	***N***	Mean (SD)	Cohen’s D	***p***
**A−T−N−** ^a^	137	2.8 (0.2)		-	138	2.2 (0.1)		-
**A−T−N+**	49	3.0 (0.2)	1.0	**0.001**	49	2.4 (0.1)	2.0	**<0.0001**
**A−T+N+**	12	3.0 (0.03)	0.8	**0.006**	12	2.6 (0.1)	4.0	**<0.0001**
**A+T−N−**	32	2.9 (0.2)	0.5	0.509	33	2.2 (0.1)	0.0	0.107
**A+T−N+**	20	2.9 (0.2)	0.5	0.062	20	2.4 (0.1)	2.0	**<0.0001**
**A+T+N+**	6	3.0 (0.0)	1.4	0.125	6	2.5 (0.1)	3.0	**<0.0001**

^a^ A−T−N− was the reference group

For CSF log(Ng) concentrations, we found that those with A−T−N+, A−T+N+, A+T−N+, and A+T+N+ (i.e., all groups with N+) had higher concentrations of CSF Ng than A−T−N−, all *p*-values <0.0001 (Fig. 2B, Table 2).

We performed an ANOVA that showed differences between the ATN groups in log(NfL) (*p*=0.002, F=3.98, DF=5) and log(Ng) (*p*<0.0001, F=54.8, DF=5).

The CSF log(NfL) concentrations between A/T/N groups differed significantly (*p*=0.005, F=3.44, DF=5), when using age, sex, *APOE* ε4 carriership, and stroke as covariates in an ANCOVA. CSF log(Ng) concentrations also differed significantly between A/T/N groups (*p*<0.001, F=57.7, DF=5), when using age, sex, *APOE* ε4 carriership, and stroke as covariates in ANCOVA.

In groupwise comparisons, most A/T/N groups differed significantly from each other in log(Ng) concentrations (Table 4). A−/T−/N− had significantly (*p*<0.001) lower concentrations than all other groups except A+/T−/N− (*p*=0.25), and A+/T+/N+ had significantly (*p*=0.03 or lower) higher concentrations than all other groups except A−/T+/N+ (*p*=1.0) (Table 4). N+ had significantly higher Ng concentrations than N− whereas no such relationship was seen between A+ vs A− groups (Table 2). In pairwise comparisons between ATN groups there were no differences in NfL levels (Table 3), with the exception of A−T−N+ vs A−T−N− (*p*=0.036).

**Table 3:** Pairwise comparisons between the ATN classes in the H70 participants with log(NfL) as a dependent variable

	ATN class	Mean difference	Std error	***p***-value
**A−T−N−**	**A−T−N+**	−0.109	0.036	**0.036**
	**A−T+N+**	−0.189	0.064	0.055
	**A+T−N−**	−0.026	0.043	1.000
	**A+T−N+**	−0.060	0.054	1.000
	**A+T+N+**	−0.144	0.093	1.000
**A−T−N+**	**A−T−N−**	0.109	0.036	**0.036**
	**A−T+N+**	−0.080	0.069	1.000
	**A+T−N**−	0.083	0.049	1.000
	**A+T−N+**	0.049	0.059	1.000
	**A+T+N+**	−0.035	0.095	1.000
**A−T+N+**	**A−T−N−**	0.189	0.064	0.055
	**A−T−N+**	0.080	0.069	1.000
	**A+T−N−**	0.163	0.072	0.354
	**A+T−N+**	0.129	0.080	1.000
	**A+T+N+**	0.045	0.109	1.000
**A+T−N−**	**A−T−N−**	0.026	0.043	1.000
	**A−T−N+**	−0.083	0.049	1.000
	**A−T+N+**	−0.163	0.072	0.354
	**A+T−N+**	−0.035	0.063	1.000
	**A+T+N+**	−0.118	0.097	1.000
**A+T−N+**	**A−T−N−**	0.060	0.054	1.000
	**A−T−N+**	−0.049	0.059	1.000
	**A−T+N+**	−0.129	0.080	1.000
	**A+T−N−**	0.035	0.063	1.000
	**A+T+N+**	−0.084	0.102	1.000
**A+T+N+**	**A−T−N−**	0.144	0.093	1.000
	**A−T−N+**	0.035	0.095	1.000
	**A−T+N+**	−0.045	0.109	1.000
	**A+T−N−**	0.118	0.097	1.000
	**A+T−N+**	0.084	0.102	1.000

Log(NfL) levels of the ATN groups were compared pairwise as a post hoc test in the ANCOVA analysis, which included a Bonferroni correction

**Table 4:** Pairwise comparisons between the ATN classes in the H70 participants with log(Ng) as a dependent variable

ATN class	ATN class	Mean difference	Std error	***p***-value
**A−T−N−**	**A−T−N+**	−0.179	0.017	**<0.001**
	**A−T+N+**	−0.331	0.031	**<0.001**
	**A+T−N−**	0.049	0.020	0.249
	**A+T−N+**	−0.172	0.026	**<0.001**
	**A+T+N+**	−0.326	0.045	**<0.001**
**A−T−N+**	**A−T−N−**	0.179	0.017	**<0.001**
	**A−T+N+**	−0.152	0.033	**<0.001**
	**A+T−N−**	0.228	0.023	**<0.001**
	**A+T−N+**	0.007	0.028	1.000
	**A+T+N+**	−0.147	0.046	**0.023**
**A−T+N+**	**A−T−N−**	0.331	0.031	**<0.001**
	**A−T−N+**	0.152	0.033	**<0.001**
	**A+T−N−**	0.380	0.034	**<0.001**
	**A+T−N+**	0.158	0.038	**<0.001**
	**A+T+N+**	0.005	0.052	1.000
**A+T−N−**	**A−T−N−**	−0.049	0.020	0.249
	**A−T−N+**	−0.228	0.023	**<0.001**
	**A−T+N+**	−0.380	0.034	**<0.001**
	**A+T−N+**	−0.221	0.030	**<0.001**
	**A+T+N+**	−0.375	0.047	**<0.001**
**A+T−N+**	**A−T−N−**	0.172	0.026	**<0.001**
	**A−T−N+**	−0.007	0.028	1.000
	**A−T+N+**	−0.158	0.038	**0.001**
	**A+T−N−**	0.221	0.030	**<0.001**
	**A+T+N+**	−0.153	0.049	**0.030**
**A+T+N+**	**A−T−N−**	0.326	0.045	**<0.001**
	**A−T−N+**	0.147	0.046	**0.023**
	**A−T+N+**	−0.005	0.052	1.000
	**A+T−N−**	0.375	0.047	**<0.001**
	**A+T−N+**	0.153	0.049	**0.030**

Log(Ng) levels of the ATN groups were compared pairwise as a post hoc test in the ANCOVA analysis, which included a Bonferroni correction

### NfL and Ng levels in Aβ and tau pathology

To analyze the effect of Aβ pathology, we compared A+ and A− groups (irrespective of tau status) in relation to their CSF NfL and Ng levels. Similarly, we compared T+ and T− individuals irrespective of Aβ status. There was no difference in CSF log(NfL) concentration between A+ and A− participants (2.89 vs. 2.87, *p*=0.503). Participants with tau pathology had higher levels of CSF log(NfL) than participants without pathology (2.94 vs 2.83, *p*=0.019).

There were no differences in CSF log(Ng) levels in those with amyloid pathology compared with those without amyloid pathology (2.28 vs 2.29, *p*=0.983). However, those with tau pathology had higher levels of CSF log(Ng) than those without tau pathology (2.42 vs 2.22, *p*<0.0001).

## Discussion

In this study, we assessed CSF NfL and Ng in cognitively unimpaired older adults from the Gothenburg H70 Birth Cohort studies classified according to the A/T/N scheme [27] and according to amyloid and tau pathology status. Our results indicate that both higher CSF NfL and higher CSF Ng is associated with an N+ and/or T+ classification, but that there are no significant associations between these two biomarkers and the A classification status. Subjects with an N+ classification were found to have both higher CSF NfL concentrations, as well as higher CSF Ng concentrations than subjects who were A−T−N− (the reference group). This was also true in most cases for participants with a T+ classification compared with those with a T− classification. There were no differences in either biomarker concentration between A+ and A− participants, with one small exception in the ANCOVA analysis. For Ng, we found that participants with A+/T+/N+ had higher concentrations than all other groups except for A−/T+/N+.

The canonical and scientifically well-backed-up sequence of events in AD development is laid out in the amyloid cascade hypothesis, stating that an imbalance in the production or clearance of Aβ is the instigating event in AD leading to the subsequent formation of amyloid plaques, tau tangles, and ultimately resulting in neuronal death [32]. As Aβ pathology is the earliest sign of AD development it is likely that it precedes neuronal and synaptic decay reflected by NfL and Ng. Furthermore, as we travel downstream in the amyloid cascade hypothesis, tau pathology coincides with developing cell loss, as confirmed by the significantly higher concentrations of Ng and partly for NfL in participants classified as N+ as compared to N−. Some studies have indicated CSF NfL as a suitable alternative proxy for the N-classification [18, 33]. CSF Ng can be used as a biomarker of synaptic degeneration in AD, an early event in AD pathogenesis [5, 9]. This was again corroborated by our findings as both N+ compared to N- were shown to exhibit significantly higher concentrations of CSF Ng in our cohort. Previous studies also suggest an association between CSF Ng and the presence of amyloid pathology [34]. Data in the present study somewhat contradict these findings as there was only a small difference in CSF Ng concentrations between the A+ and A− groups. However, as all our subjects were cognitively healthy, and thus were still in the preclinical stages of AD, the differences between the A+ and A− groups may be attenuated. However, we found an association between individuals positive for all three biomarkers A+T+N+ and Ng, these individuals have presumably progressed the furthest in AD pathology and may be nearest to a conversion to MCI or dementia.

Synapse loss may play a role in neurodegeneration and perhaps cognitive decline in dementia. Synapse loss and dysfunction occur in many neurodegenerative diseases [12] and can be seen early in AD [7] especially in the hippocampus [35]. The reasons behind synapse degeneration and loss is unclear [7], and there are several hypotheses relating to synapse loss. One hypothesis suggests that it could be caused by excessive synaptic pruning (a part of normal development) by the complement system, which could become reactivated in neurodegenerative conditions causing adverse effects [36]. Another hypothesis suggests that tau pathology spreads from cell to cell throughout the brain, but how this relates to neurodegeneration and disease progression is less clear [37]. Tau seems to have a function at the synapse and may be involved in synaptic dysfunction in dementia [35, 38]. Another possible explanation might be that neuron damage can occur without prior formation of amyloid plaques, as has been suggested by the oligomer hypothesis [39]. Aβ oligomers are believed to be neurotoxic and to be present early in AD and may cause synaptic dysfunction or loss [40–42]. The oligomer hypothesis suggests that oligomers cause memory loss in AD by disruption of synaptic plasticity [40]. There is some evidence that oligomers are elevated in the brains of AD patients and AD mouse models [41–43] where they seem to surround cortical neurons and bind to synapses [41]. One group used transgenic mouse models to show that expression of APP (amyloid precursor protein) in neurons resulted in a synaptic loss, which correlated with Aβ levels, but did not require Aβ plaques [44]. Another study in mice showed that memory loss was reversible after treatment with an anti-Aβ monoclonal antibody, without reducing brain amyloid burden, suggesting that this could possibly be due to clearance of soluble Aβ in the brain [45]. It has been suggested that oligomers may be the cause of the early memory disturbances that can be seen in AD, by affecting long-term potentiation and synaptic plasticity [39, 42]. Oligomers can also affect other events important in AD pathology such as oxidative stress, neuroinflammation hyperphosphorylation of tau, and synaptic or neuronal death [40, 46, 47]. Since our results showed that the Ng levels were high in participants both with and without amyloid pathology this could perhaps suggest that other factors than amyloid plaques are driving the synaptic damage. One possibility could be that this driving factor might be oligomers, given their relation to synaptic pathology.

### Strength and limitations

We have chosen a study design where we examined the relationship between the ATN system and the biomarkers NfL and Ng, using T-tau as the “N” biomarker rather than NfL, although it could be argued that NfL could have been used in the N category instead. In our analysis we wanted to study the traditional ATN classification, with T-tau concentrations representing N, to enhance knowledge on the relationship between the newer CSF biomarkers NfL /Ng and the hallmark AD biomarkers [27]. Furthermore, since NfL reflects degeneration of particularly large-caliber axons in white matter, which is only one facet of the neuronal damage in AD, T-tau is arguably better suited as the N-marker for the purposes of this study, as it is more specifically correlated to cortical neuronal dysfunction in AD which is an early feature of the disease continuum. The participants of our cohort were cognitively unimpaired and undiagnosed, and the aim of the study was to investigate the synaptic and axonal damage caused by preclinical AD specifically. The biomarkers NfL and Ng correlated weakly with each other, and Ng was highly correlated with T-tau and P-tau. Both NfL and Ng are related to neurodegeneration, although different aspects of the process and might differ in relation to the disease stage because of this. The order of magnitude of the correlations is in keeping with previously published correlation results [18].

Some of the strengths of the study include the population-based sample with CSF data from a relatively large number of individuals. However, there were a small number of participants in some of the ATN groups, which may lead to lowering of statistical power. Some ATN patterns are hard to explain in terms of AD pathology, A−T+N− for example, as individuals with no amyloid pathology normally do not present with NFTs. A possible explanation for this is that these participants might be misclassified due to biomarker assessment errors, which highlights another weakness of this study, namely the vulnerability of dichotomizing biomarker readouts into healthy and pathological classifications. However, as all biomarker measurements were carried out at the same world-renowned laboratory, by accredited personnel, and with well-proven commercial test kits this weakness is attenuated as far as possible. Furthermore, the lack of follow-up information indicating which types of pathology were developed by each participant is also a weakness of this study, e.g*.*, stable MCI, vascular dementia, dementia with Lewy bodies, frontotemporal dementia, Parkinson’s disease dementia, and other neurodegenerative disease processes behave differently regarding biomarker patterns. It should also be noted that Aβ, T-tau, P-tau, and Ng are all known to be associated with AD pathology and each other, making it difficult to establish causal relationships between them. NfL, on the other hand, also shows an AD-independent association with neurodegeneration, making it slightly different than the other biomarkers, a result that agrees with earlier findings [48].

In conclusion, this study showed that concentrations of especially CSF-Ng but also CSF-NfL were higher in cognitively healthy subjects classified as N+ and/or T+ according to A/T/N criteria. This finding corroborates the literature proposing CSF NfL and Ng as early biomarkers of neurodegeneration and synaptic dysfunction in AD. However, further studies with longer follow-ups and detailed characterizations of symptom and biomarker trajectories are needed.

## Data Availability

The datasets used and/or analyzed during the current study are available from the corresponding author on reasonable request.
